# Monitoring Soil Copper in Urban Land Using Visibale and Near-Infrared Spectroscopy with Spatially Nearby Samples

**DOI:** 10.3390/s24175612

**Published:** 2024-08-29

**Authors:** Yi Liu, Tiezhu Shi, Zeying Lan, Kai Guo, Chao Yang, Yiyun Chen

**Affiliations:** 1 School of Public Administration, Guangdong University of Finance & Economics, Guangzhou 510320, China; 2 State Key Laboratory of Subtropical Building and Urban Science & Guangdong–Hong Kong-Macau Joint Laboratory for Smart Cities & MNR Key Laboratory for Geo-Environmental Monitoring of Great Bay Area, Shenzhen University, Shenzhen 518060, China; 3 School of Management, Guangdong University of Technology, Guangzhou 510520, China; 4 School of Geography and Remote Sensing, Guangzhou University, Guangzhou 510006, China; 5 School of Resource and Environmental Sciences, Wuhan University, Wuhan 430079, China; 6 Key Laboratory of Geographic Information System of the Ministry of Education, Wuhan University, Wuhan 430079, China; 7 Hubei Luojia Laboratory, Wuhan 430079, China

**Keywords:** soil spectroscopy, proximal sensing, heavy metal, spatial analysis

## Abstract

Soil heavy metal contamination in urban land can affect biodiversity, ecosystem functions, and the health of city residents. Visible and near-infrared (Vis-NIR) spectroscopy is fast, inexpensive, non-destructive, and environmentally friendly compared to traditional methods of monitoring soil Cu, a common heavy metal found in urban soils. However, there has been limited research on using spatially nearby samples to build the Cu estimation model. Our study aims to investigate how spatially nearby samples influence the Cu estimation model. In our study, we collected 250 topsoil samples (0–20 cm) from China’s third-largest city and analyzed their spectra (350–2500 nm). For each unknown validation sample, we selected its spatially nearby samples to construct the Cu estimation model. The results showed that compared to the traditional method ($R_{p}^{2}$ = 0.75, RMSEP = 8.56, RPD = 1.73), incorporating nearby samples greatly improved the model ($R_{p}^{2}$ = 0.93, RMSEP = 4.02, RPD = 3.89). As the number of nearby samples increased, the performance of the Cu estimation model followed an inverted U-shaped curve—initially increasing and then declining. The optimal number of nearby samples is 125 (62.5% of the total), and the mean distance between validation and calibration samples is 17 km. Therefore, we conclude that using nearby samples significantly enhances the Cu estimation model. The optimal number of nearby samples should strike a balance, covering a moderate area without there being too few or too many.

## 1. Introduction

Soil heavy metal contamination poses challenges to several United Nations Sustainable Development Goals (SDGs), including good health and well-being, sustainable ecosystems and cities, and climate change regulation [1,2]. Soil heavy metal contamination can directly affect biodiversity and ecosystem functions [3]. Moreover, soil heavy metal contamination in urban areas can affect the health of city residents through food chains, drinking water, and direct contact with soil in greenspaces [4]. Soil heavy metal contamination comes from both human activities and natural sources. Human activities include vehicle emissions, industrial processes, and poor waste management, while natural sources include metal-rich rock weathering [5]. Urban soil is more influenced by human activities than natural soil, especially due to rapid industrialization and urbanization, particularly in developing countries [6,7]. Thus, it is urgent to monitor soil heavy metal contamination in urban areas.

Traditional methods of measuring soil heavy metals are expensive and time-consuming [8,9]. Copper (Cu) is a common heavy metal found in urban soil [10]. The traditional method of Cu estimation is inductively coupled plasma mass spectrometer (ICP-MS) [11]. This method requires sample preparation and chemical preprocessing, making it time-consuming and costly [12,13]. In recent years, visible and near-infrared (vis-NIR) spectroscopy has been developed as an alternative method for estimating heavy metal content in soil [14,15,16,17]. This method is fast, cheap, non-destructive, and environmentally friendly [18,19,20].

When using vis-NIR spectroscopy, calibration samples were first used to build a Cu estimation model. Then, new samples were tested with this built model to predict their Cu content [21]. Covering a large area would generate many samples and increase their diversity [22]. Thus, it is challenging for the built model to be suitable for various conditions, such as different parent materials, soil types, land use, and landscapes [23]. One possible way to decrease the diversity is by considering spatial similarity [24]. In other words, the nearby samples of a validation sample can be used to build the model and predict the Cu content of that validation sample. In fact, samples that are close together are more likely to share a similar Cu estimation model than those that are far apart [25,26]. Thus, it is important to consider spatially nearby samples when building a Cu estimation model.

Early researchers have studied the spatial similarities in estimating soil properties. Viscarra Rossel et al. (2024) [27] assessed geographic similarities and pointed out that samples under similar pedo-climatic conditions benefit the local soil property estimation. Song et al. (2024) [28] considered geographical stratification and achieved the highest accuracy in soil total nitrogen estimation. Khosravi et al. (2024) [29] used geographical and texture-based stratification strategies to enhance the soil organic carbon prediction. Hong et al. (2023) [30] found that considering land use in more homogeneous classes led to better soil inorganic carbon predictions. These researchers found that similarity in position is important for estimating soil properties [31,32,33]. However, previous studies on spatial similarity did not use samples from nearby areas; instead, they used methods like geographic stratifications.

Spatially nearby samples mean using the surrounding samples to predict the Cu content of a specific sample. Instead of focusing solely on nearby samples, many researchers have explored geographic zones, geographic subsets, spatial dependence, or landscapes [34,35,36]. Shi et al. (2015) [37] used soil geographical zoning to account for similarities in soil-forming conditions. Vohland et al. (2022) [38] considered geographical subsets to improve the performance of the soil organic carbon estimation model. Geographic zones or stratifications mean dividing the samples into groups based on their location. This approach increases similarities within each area, but it does not ensure that each sample is close to its nearest samples. However, in theory, nearby samples are more suitable and preferred [27,39]. Focusing on similar spatial or close distances makes samples share more common characteristics, such as spectral shapes and environmental factors [40]. Summerauer et al. (2021) [41] found that the nearest neighbor samples are a suitable method. Dorantes et al. (2022) [42] reported that reducing the geographic extent of a calibration model may reduce the spectral feature space. Thus, our study focuses on spatially nearby samples that are located to each other instead of using geographic zones or stratifications, as many previous researchers have done. We investigated how these nearby samples influence soil Cu estimation. Moreover, how many spatially nearby samples are suitable for the soil Cu estimation model? Is it a case of “the more, the better” for performance? However, there is less research on the optimal number of spatially nearby samples required for soil Cu estimation.

To overcome this challenge, we aim to address two gaps in current soil spectroscopy research: (i) using spatially nearby samples to estimate soil Cu in urban land by vis-NIR spectroscopy; (ii) determining the optimal number of spatially nearby samples for the Cu estimation model.

## 2. Materials and Methods

### 2.1. Study Area and Sample Collection

**Figure 1:**
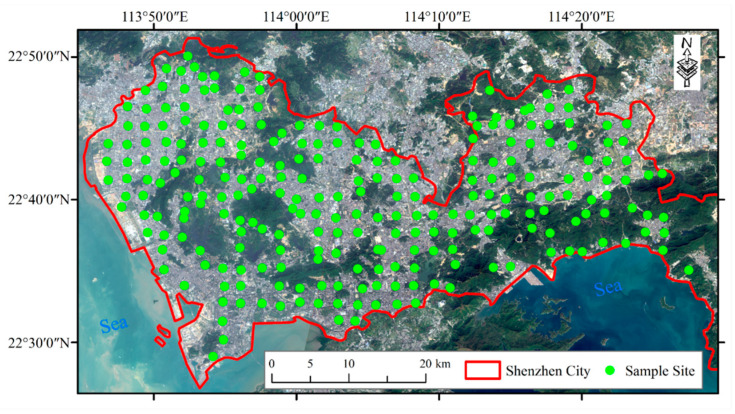
Location of the sampling sites.

Our study area is Shenzhen City, located in southeast China (113°46′ E to 114°37′ E and 22°27′ N to 22°52′ N), as shown in Figure 1. This city is the 3rd-largest in China and the 10th-largest in the world. In 1979, most of this city was agricultural land, with a population of 3.14 million people. However, following China’s reform and opening-up policy in 1978, the city experienced rapid industrialization and urbanization, growing to a population of 17.79 million people by 2023. The city’s GDP has soared to $482 billion, making it one of the most developed cities in the world. Previous studies have indicated that intensive human activities, such as industrial wastewater, the use of fertilizers and pesticides, vehicle emissions, and household garbage, have resulted in soil contamination by heavy metals [43,44].

This city is located near the sea and close to the Tropic of Cancer (23.5° N). It has an average temperature of 22.4 °C, making it a warm place. The average annual rainfall is 1933 mm, with most of the rain falling in summer, which may cause soil erosion. As classified by the Genetic Soil Classification of China (GSCC), the main soil types in this area are latosolic red soils, red soils, yellow soils, paddy soils, and coastal solonchaks [45]. According to the World Reference Base for Soil Resource (WRB), the main soil types are acrisols, cambisols, anthrosols, and solonchaks [46]. The city’s unique natural conditions and extensive human activities make it an ideal place to study soil heavy metal contamination.

The study area was divided into grids measuring 2 × 2 km, and a sampling site was randomly chosen from each grid. At each site, we removed surface cover or plants and collected 1.5 kg of topsoil from a depth of 0–20 cm during five sampling campaigns [43,47]. However, accessing some grids was difficult due to the city’s hilly terrain, as shown in Figure 1. We made sure to avoid artificial deposits, such as rubbles, concrete debris, and waste. In total, we collected 250 samples in November 2016, and their positions were recorded using a GPS receiver. We also noted the land use, vegetable cover, and landform at each site.

### 2.2. Spectral Measurement and Chemical Analysis

**Figure 2:**
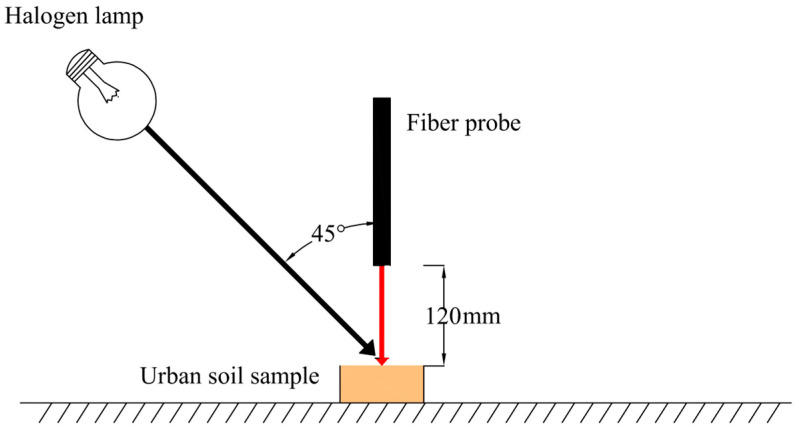
Equipment setup for spectral measurements.

In the lab, the samples were air-dried and ground until they could pass through a 2 mm sieve. Then, the samples were divided into two parts: one for spectral analysis and the other for chemical analysis. The spectra were obtained using an ASD FieldSpec^®^ 3 portable spectro-radiometer (Analytical Spectral Devices Inc., Boulder, CO, USA), which has a spectral range of 350–2500 nm [48]. The spectra scan was performed in a dark room using a halogen lamp positioned at a 45° angle above the sample (Figure 2). A fiber probe was placed 12 cm directly overhead at a 90° angle. Before measurement, the spectrometer was calibrated with a Spectralon^®^ panel that had 99% reflectance. Each sample was scanned 10 times, and the results were averaged. The Cu content was analyzed using the diethylenetriamine penta-acetic acid method and measured with ICP-OES [11,49].

### 2.3. Spatially Nearby Samples

**Figure 3:**
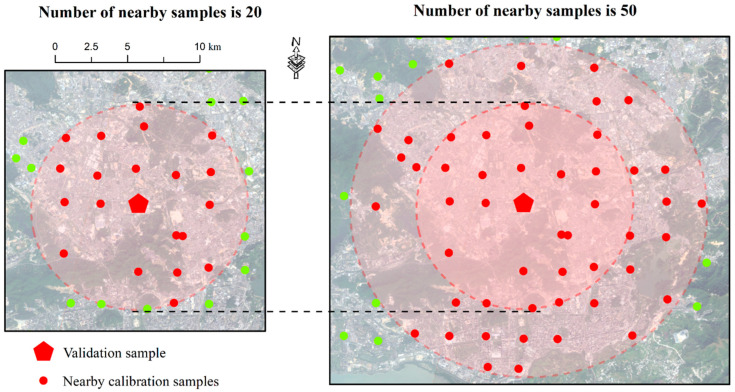
A validation sample with 20 and 50 spatially nearby samples used for building the Cu estimation model. The green circles denote samples that were not selected as nearby samples.

**Figure 4:**
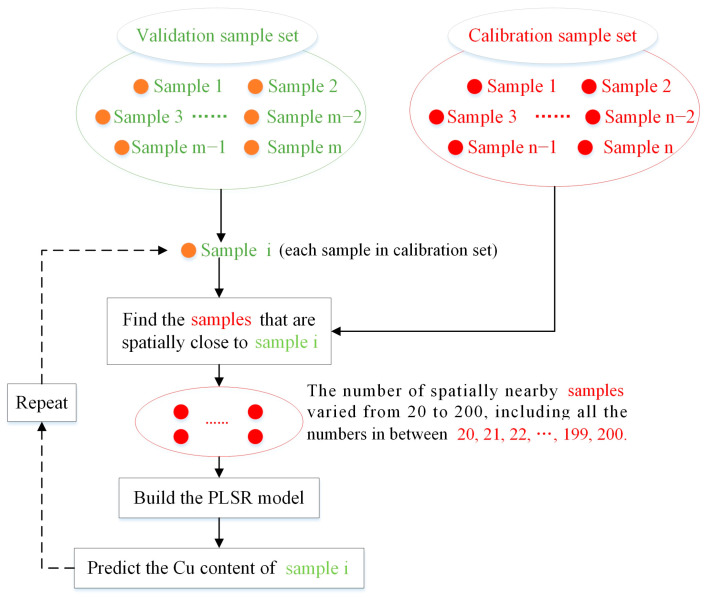
Flowchart of using spatially nearby samples.

In theory, spatially nearby samples share many conditions. Therefore, our study aims to use these samples to build a Cu estimation model. For each unknown validation sample, we selected its surrounding samples based on geographic distance to construct the Cu estimation model. For example, as shown in Figure 3, we selected 20 and 50 nearby samples to predict the Cu content of the validation sample. For each validation sample, a set of nearby samples was selected to build a Cu estimation model (Figure 4). This study involved 50 validation samples, resulting in the creation of 50 Cu estimation models.

To study the influence of nearby samples on the Cu estimation model, we varied the number of nearby samples from 20 to 200 (Figure 4). Our calibration set contains a total of 200 samples. Traditionally, researchers would use all 200 calibration samples to build one model to predict the Cu content of the 50 validation samples. However, our study focuses solely on using nearby samples. The number of nearby samples varied from 20, 21, 22, …, up to 200.

### 2.4. Model Calibration

A total of 250 samples were divided into 200 calibration samples and 50 validation samples. The 20%/80% split is commonly used by other researchers [13,50]. The 50 validation samples were selected based on their Cu content. Samples were ordered in ascending Cu content, and every fifth sample was chosen. This method ensured that the validation sample set evenly covered the range of Cu content of this city, making it suitable for future new samples from this city. 

Partial least squares regression (PLSR) was used to build the model. Although many researchers have recently used deep learning methods like random forest (RF) [51], our study focuses on spatially nearby samples and uses the most commonly used method, PLSR [52]. PLSR first projects the spectra into a low-dimensional space, where multiple regression is then performed. Based on previous research [21], the Cu estimation model does not benefit from or require spectral pretreatment, so we did not apply any. 

As mentioned in Section 3.3, not all 200 calibration samples were used at once. For each validation sample, a specific number of nearby samples were selected to form the calibration set. The calibration set was then used to calibrate the PLSR model and predict the Cu content of the validation sample. The number of latent variables was determined using leave-one-out cross-validation (LOOCV). The PLSR was conducted using the PLS_toolbox (Eigenvector Research, Inc., Manson, WA, USA) within the MATLAB environment (The MathWorks, Inc., Natick, MA, USA).

### 2.5. Model Performance

The 50 validation samples were used to test the performance of the PLSR model. Several common indicators were used to assess the model’s performance: coefficient of determination in prediction ($R_{p}^{2}$), root mean square error of prediction (RMSEP), and residual predictive deviation (RPD). These indicators were calculated as follows:

(1) $$R_{p}^{2}=\sqrt{1-\frac{\sum_{i=1}^{n}\left(y_{i}-{\hat{y}}_{i}\right)}{\sum_{i=1}^{n}\left(y_{i}-\bar{y}\right)}}$$

(2) $$RMSEP=\sqrt{\frac{\sum_{i=1}^{n}\left(y_{i}-{\hat{y}}_{i}\right)}{n}}$$

(3) $$RPD=\frac{SD}{RMSEP}$$

where *n* is the number of validated samples, and $y_{i}$ and ${\hat{y}}_{i}$ are the measured and predicted Cu values of *i*th sample in the validation set. $\bar{y}\;$ is the average measured Cu value. SD is the standard deviation of measured Cu values.

## 3. Results

### 3.1. Descriptive Statistics of Soil Samples

**Figure 5:**
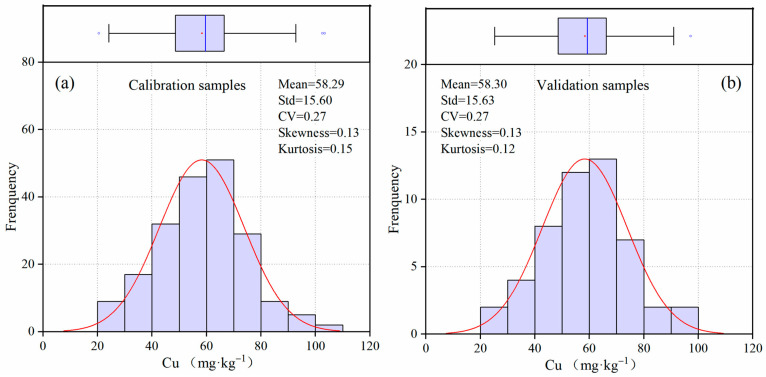
Boxplot and histogram of Cu content for calibration samples (**a**) and validation samples (**b**). Repoint (·) denotes the mean value. The blue line (|) denotes the median value. Hollow circle (○) denotes the outliers. The black box denotes the interquartile range.

**Figure 6:**
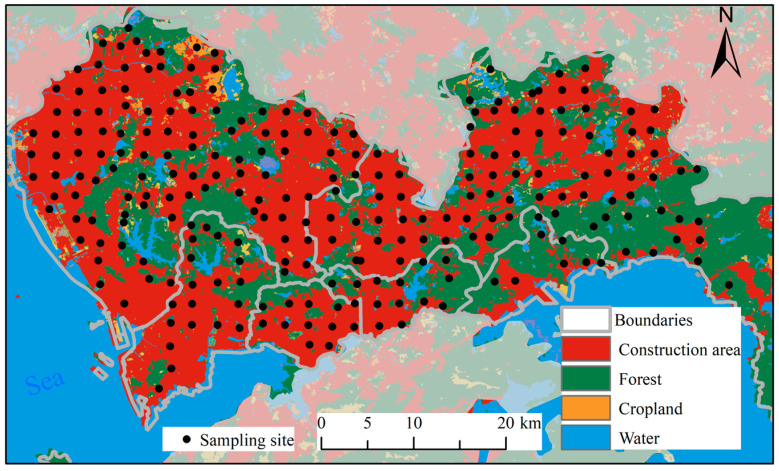
Land use and sample distribution in Shenzhen city.

The Cu content ranged from 20.45 to 103.24 mg·kg^−1^, with a mean value of 58.29 mg·kg^−1^ (Figure 5 and Table 1). The mean Cu value was three times higher than the background value (17 mg·kg^−1^), indicating that extensive human activities have caused significant Cu pollution in the soil. According to the pollution level (36 mg·kg^−1^) [20], 230 samples showed different degrees of Cu pollution, with a pollution rate of 92%. The severe pollution in Shenzhen may be attributed to the city’s rapid urbanization (Figure 6). As shown in Figure 6, much of the city’s land has been converted from cropland to construction areas. With a population of 17.79 million and industrial activities amounting to $482 billion, soil heavy metal pollution has become an urgent environmental issue.

The coefficient of variation (CV) was 0.27, indicating a medium level of variability (0.1 < CV < 1.0). The skewness (0.13) and kurtosis (0.12) were close to zero, indicating a normal distribution. For the calibration and validation sets, Levene’s test confirmed significant homogeneity at the 0.05 significance level (*p* = 0.99). This was also evidenced by Figure 5, where the boxplot and histogram of calibration and validation sets were very similar.

### 3.2. Estimation Performance of Cu Models without Considering Spatially Nearby Samples

**Figure 7:**
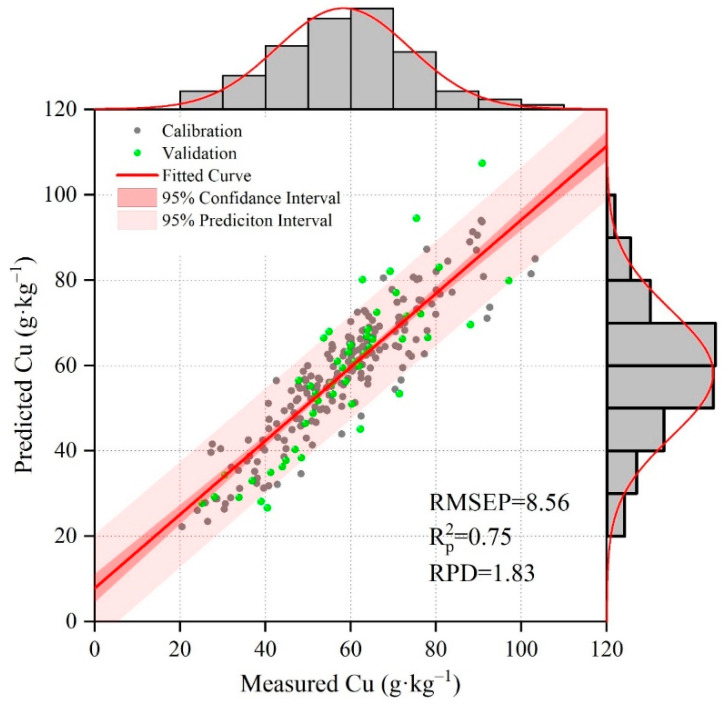
Soil Cu content between predicted and measured values using spectroscopy models without considering spatially nearby samples. $R_{p}^{2}$ denotes coefficient of determination in prediction. RMSEP denotes the root mean square error of prediction. RPD denotes the residual predictive deviation.

When not considering spatially nearby samples, the performance of the Cu estimation was acceptable. The $R_{p}^{2}$ was 0.75, RMSEP was 8.56 mg·kg^−1^, and RPD was 1.73 (Figure 7). Most samples were located close to the fit curve. Compared to the 1:1 line, the slope of the fit curve is less than 1 (less than a 45° angle), indicating that low Cu content was overestimated while high Cu content was underestimated. According to the histogram, the measured and predicted Cu content were similar, showing that the model did not change the Cu distribution. Given that our study area was a large city with 17.79 million people and 1997 km^2^, these three indicators showed that the Cu estimation model was satisfactory.

### 3.3. Estimation Performance of Cu Models with Spatially Nearby Samples

**Figure 8:**
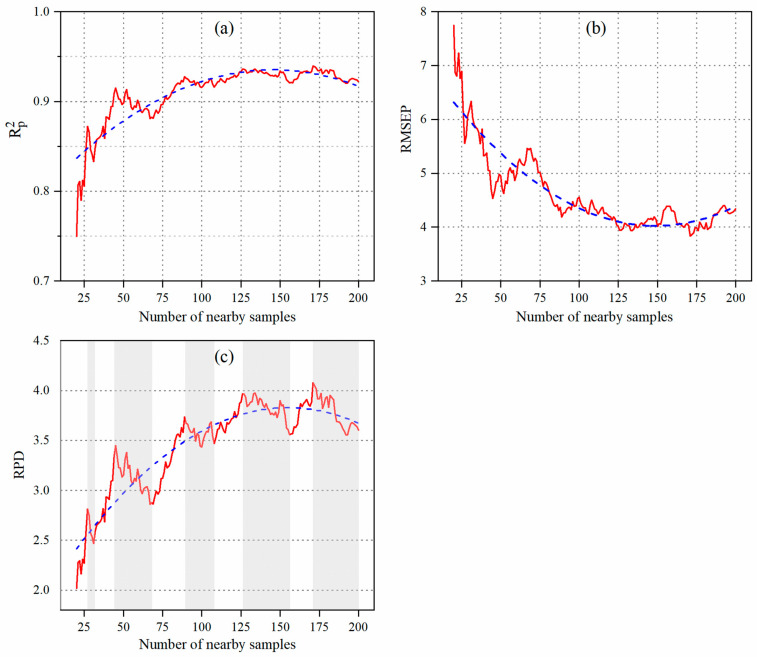
Performance of soil Cu estimation model considering different numbers of spatial nearby samples. (**a**) $R_{p}^{2}$, denotes coefficient of determination in prediction. (**b**) RMSEP, denotes the root mean square error of prediction. (**c**) RPD, denotes the residual predictive deviation. The dotted blue line is the fitting line.

The $R_{p}^{2}$ increased as more spatially nearby samples were selected (Figure 8a and Table 2). With only 20 nearby samples, the $R_{p}^{2}$ was 0.75. It then quickly rose to 0.90 with 50 samples. Therefore, between 20 and 50 samples, nearby samples were most beneficial for improving the Cu estimation model. From 50 to 70 samples, the $R_{p}^{2}$ decreased slowly to 0.88. When there were more than 70 samples, the $R_{p}^{2}$ increased very slowly, reaching 0.93 at 125 samples. Beyond 125 samples, the $R_{p}^{2}$ stabilized with minor fluctuations and tended to decrease. Thus, selecting more than 125 nearby samples did not significantly benefit the Cu estimation model and may have even degraded the model.

The RMSEP decreased as more spatially nearby samples were selected (Figure 8b). With 20 nearby samples, the RMSEP was 7.75 mg·kg^−1^. It then quickly dropped to 4.90 mg·kg^−1^ with 50 samples, showing the most significant decrease in RMSEP between 20 and 50 nearby samples. Afterward, it rose to 5.46 mg·kg^−1^ with 70 samples. From 70 to 125 samples, the RMSEP slowly decreased to 4.04 mg·kg^−1^. Beyond 125 samples, it remained stable with minor fluctuations and tended to increase. Thus, using more than 125 nearby samples did not improve the RMSEP and could even increase it.

The RPD increased in a wave-like pattern as more spatially nearby samples were selected (Figure 8c). There were five peaks in the RPD curve at 28, 43, 80, 125, and 172 samples, with RPD values of 2.81, 3.09, 3.24, 3.88, and 4.08, respectively. The RPD increased before each peak and then decreased afterward. The wave-like pattern suggested that initially, adding nearby samples improved the model, but adding too much eventually degraded it. Therefore, it is crucial to determine the right number of nearby samples—not too few and not too many.

The RPD performed similarly to $R_{p}^{2}$ and RMESP in overall (Figure 8c). According to the fit curve (blue dotted line in Figure 8c), the RPD generally rose from 20 to 125 samples and then stabilized with a tendency to decrease. At 20 samples, the RPD was 2.01, and at 125 samples, it was 3.88.

In total, the model’s performance improved significantly as the number of samples increased from 20 ($R_{p}^{2}$ = 0.75, RMSEP = 7.75 mg·kg^−1^, RPD = 2.01) to 50 ($R_{p}^{2}$ = 0.90, RMSEP = 4.90 mg·kg^−1^, RPD = 3.14). However, its performance decreased from 50 to 70 samples ($R_{p}^{2}$ = 0.88, RMSEP = 5.46 mg·kg^−1^, RPD = 2.86). Between 70 and 125 samples, the performance increased gradually ($R_{p}^{2}$ = 0.93, RMSEP = 4.02 mg·kg^−1^, RPD = 3.89). Beyond 125 samples, the model’s performance stabilized with minor fluctuations and a tendency to degrade.

Compared to the model that did not consider spatially nearby samples ($R_{p}^{2}$ = 0.75, RMSEP = 8.56 mg·kg^−1^, and RPD = 1.73), using nearby samples significantly improved the Cu estimation model. With 20 nearby samples, the $R_{p}^{2}$ was 0.75, RMSEP was 7.75 mg·kg^−1^, and RPD was 2.01, indicating similar or better performance than using all 200 samples in the traditional way. As more nearby samples were selected, $R_{p}^{2}$ increased, RMSEP decreased, and RPD increased, resulting in superior performance than the traditional Cu estimation model. With 125 nearby samples ($R_{p}^{2}$ = 0.93, RMSEP = 4.02 mg·kg^−1^, and RPD = 3.89), the Cu estimation model greatly outperformed than traditional model ($R_{p}^{2}$ = 0.75, RMSEP = 8.56 mg·kg^−1^, and RPD = 1.73).

**Figure 9:**
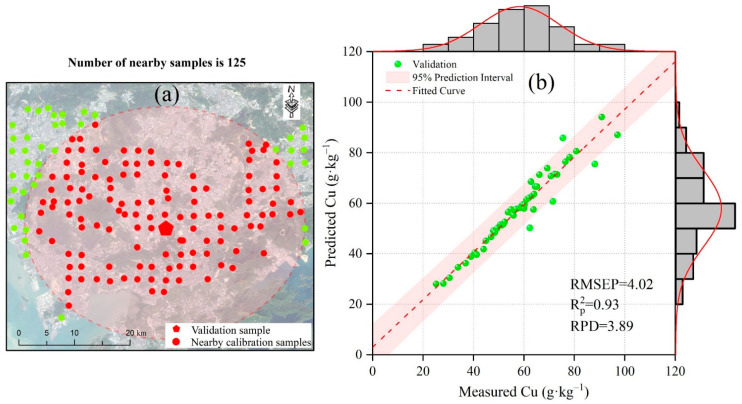
Performance of soil Cu estimation model when the number of spatial nearby samples is 125. $R_{p}^{2}$ denotes coefficient of determination in prediction. RMSEP denotes the root mean square error of prediction. RPD denotes the residual predictive deviation. (**a**) The selected 125 nearby samples. (**b**) The performance of the Cu estimation model.

As shown in Figure 8, the Cu estimation model improved until it reached 125 samples. Beyond 125 samples, its performance stabilized with minor improvement and even a slight tendency to degrade. Therefore, the number of 125 nearby samples was studied specifically (Figure 9). In Figure 9b, the Cu estimation model demonstrated very high accuracy ($R_{p}^{2}$ = 0.93, RMSEP = 4.02 mg·kg^−1^, and RPD = 3.89). Most samples were located very close to the fit curve, indicating the model’s low prediction errors.

As shown in Figure 9a, the area covered by the 125 samples was very moderate—not too large to cover the entire city and not too small to cover just a tiny part of it. These findings were consistent with the “wave-like pattern” discussed in the RPD section: it is crucial to determine the right number of nearby samples—not too few and not too many. Thus, the optimal number of nearby samples should strike a balance, being neither too large nor too small, and should cover a moderate area.

## 4. Discussion

### 4.1. Estimation of Soil Cu Content in Urban Land by Vis-NIR Spectroscopy

In recent years, many researchers have used vis-NIR spectroscopy to measure soil heavy metal content. Karami et al. (2024) [53] estimated manganese (Mn), iron (Fe), nickel (Ni), zinc (Zn), and Cu using vis-NIR spectroscopy. Zhou et al. (2024) [54] estimated soil Mn with an $R_{p}^{2}$ of 0.76. Krzebietke et al. (2023) [17] estimated soil cadmium (Cd), Cu, lead (Pb), Ni, chromium (Cr), Zn, Mn, and Fe. This shows that vis-NIR spectroscopy is a feasible method for estimating soil heavy metals. Our study successfully estimated soil heavy metal Cu in urban land with high accuracy ($R_{p}^{2}$ = 0.93, RMSEP = 4.02 mg·kg^−1^, and RPD = 3.89, Figure 9b). Similar results have been reported by others, with $R_{p}^{2}$ values of 0.92 [17], 0.90 [55], and 0.82 [53]. However, these studies covered a very narrow range of Cu content (1.12–6.14 mg·kg^−1^, 0.89–6.18 mg·kg^−1^, 0.50–2.70 mg·kg^−1^). Our study covered a much broader range of Cu content (20.45 to 103.24 mg·kg^−1^, Figure 5) and included more samples. In contrast, some researchers obtained much worse results, such as $R_{p}^{2}$ values of 0.5 [56], 0.26 [8], and 0.01 [57]. This may be due to the large range of Cu range (34.25–91.40 mg·kg^−1^, 69.3–119 mg·kg^−1^, 2.7–780 mg·kg^−1^). These studies also covered a large area, with samples located 80 to 200 km apart. In contrast, Khosravi et al. (2020) [16] focused on a small area (about 1 km^2^) with a broader range of Cu content (322–5099 mg·kg^−1^) and achieved a high $R_{p}^{2}$ value of 0.86. Thus, when covering a large geographic area and range of Cu content, it is feasible to consider spatially nearby samples, like in our study, to improve the Cu estimation model.

**Figure 10:**
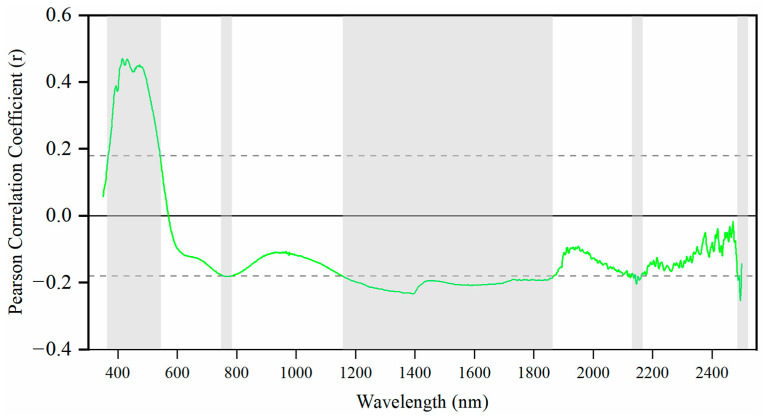
Correlation between Cu concentration and spectral wavelengths from 350 to 2500 nm. The blue line denotes the Pearson correlation coefficient. The dotted line denotes the threshold for important wavelengths.

The most important wavelengths for PLSR were 368–541 nm, 755–784 nm, 1156–1804 nm, 2143–2161 nm, and 2486–2498 nm, showing a high correlation coefficient (Figure 10). Specifically, wavelengths such as 368–541 nm, 755–784 nm, 1750–1804 nm, and 2143–2161 nm were identified as crucial for soil organic matter [58,59]. Previous studies have noted significant overlap in important wavelengths between soil heavy metals and soil organic matter [8,9]. This overlap is primarily due to Cu’s minimal or nonexistent response in the vis-NIR spectroscopy region, depending instead on other chemical bonds such as ${Fe}_{2}O_{3}$, O–H, and C–H [60]. Many researchers have found the relationship between heavy metals and soil organic matter [15]. The 1400 nm was likely attributed to water. Additionally, 2486–2498 nm was relative to clay minerals and oxides [53].

### 4.2. The Influence of Spatially Nearby Samples on Soil Cu Estimation Model

Compared to traditional methods that did not consider spatially nearby samples, taking nearby samples into account greatly improved the Cu estimation model. The $R_{p}^{2}$ increased from 0.75 to 0.93, the RMSEP decreased from 7.75 to 4.02 mg·kg^−1^, and the RPD increased from 1.73 to 3.89 (Figure 7 and Figure 8). The improvement was clear. This is because nearby samples have more in common than distant samples, making the model more reliable. In geography, distance plays an important role in the similarity of soil properties between samples [61]. Many studies suggest using nearby samples [27,40,62], but few actually do this. This may be due to the complexity of identifying each sample’s nearby samples and building a specific model for the sample. Our study took several weeks to complete the calculations, which is much longer than using a constant model that does not consider nearby samples.

**Figure 11:**
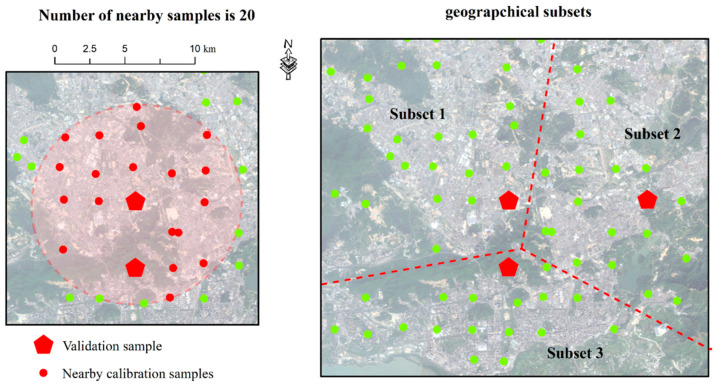
Examples of spatially nearby samples and geographic subsets.

When studying the spatial influence on soil properties, some researchers used geographical zones [37] or geographical subsets [38]. These methods divide samples into several areas, as shown in Figure 11, and then build a model for each subset. This approach reduces sample diversity and improves the model. It is simpler and requires less calculation time than our study. However, there were two main drawbacks: (i) a validation sample may be located on the edge of the subset, like Subset 1 and Subset 3 in Figure 11, making it less similar to other samples in that subset, and (ii) the number of samples in each subset can vary greatly, with some having too few and others having too many. Our study overcomes these limitations perfectly. Each validation sample is located centrally and surrounded by similar samples (Figure 11). Additionally, the number of nearby samples is consistent for all validation samples. Some research also used subsets based on landscape [34], soil type [23,63,64], or parental materials [65], which is similar to using geographical subsets. It is clear that using spatially nearby samples has many advantages.

The Cu estimation model’s performance varied based on the number of spatially nearby samples selected (Figure 8). Significant improvements were observed when increasing the nearby samples from 20 to 70, but the rate of improvement slowed between 70 and 125 samples and remained stable above 125. Therefore, the benefit of using spatially nearby samples is greatest when their number is small but diminishes with too many nearby samples. Other researchers have also found that increasing the calibration set size beyond a certain point does not improve the model performance [14,66] and can even lead to a decrease [37,67]. Thus, it is crucial to determine the optimal number of nearby samples.

**Figure 12:**
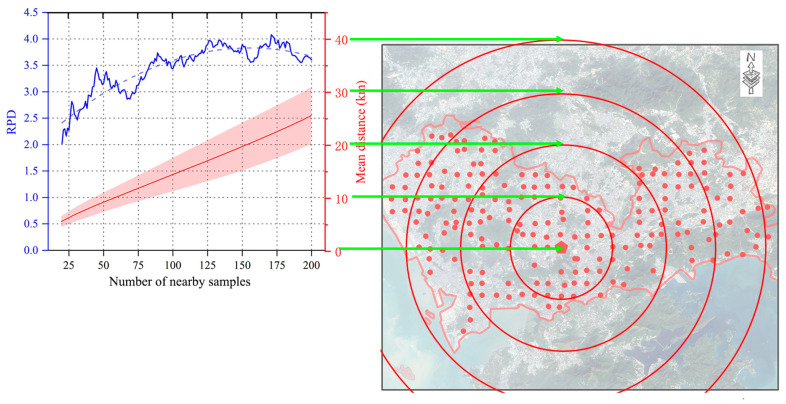
The mean distance between validation and calibration samples when selecting different numbers of nearby samples.

The change in the number of nearby samples reveals a significant factor: the spatial distance between validation and calibration samples (Figure 12). Figure 12 illustrates how the mean distance between these samples varies with the number of nearby samples, increasing linearly. This trend results from our sampling strategy, which divides the study area into 2 × 2 km grids (Section 2.1). However, this linear increase in distance does not correspond to a linear increase in model performance. Instead, the model performance follows an inverted U-shaped curve—initially increasing and then decreasing. This indicates that spatial influence is strong at closer distances but diminishes over longer distances [68,69]. Therefore, it is important to investigate the limited range of spatial influence.

**Figure 13:**
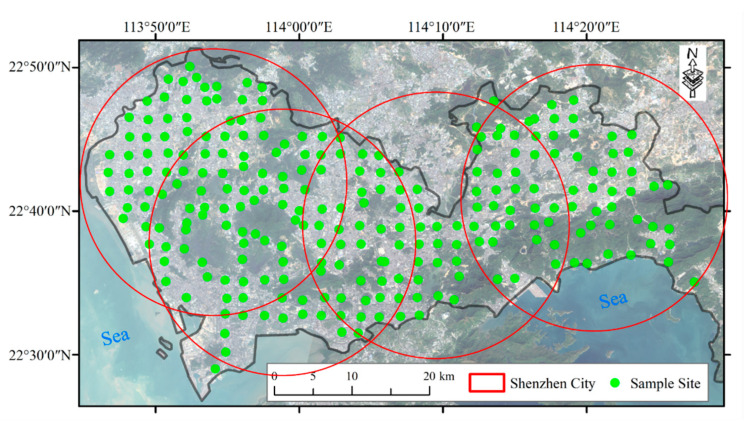
The mean distance (17 km) between the validation and calibration sample when selecting 125 nearby samples. The red circle has a radius of 17 km.

The optimal number of nearby samples is 125, which is 62.5% of the total calibration samples (Figure 9). The Cu estimation model with 125 nearby samples has high accuracy ($R_{p}^{2}$ = 0.93, RMSEP = 4.02 mg·kg^−1^, RPD = 3.89). Too few samples are insufficient to build a reliable model, while too many degrade the model’s performance [14,67]. When selecting 125 nearby samples, the mean distance between validation and calibration samples is 17 km (Figure 13). The red circle with a 17 km radius adequately covers the study area, being neither too big nor too small. Therefore, this distance is moderate and appropriate for the study area. In summary, the number of nearby samples should be balanced to cover a moderate area without there being too few or too many.

Our studies greatly improve the soil Cu estimation by using spatially nearby samples. However, there are still limitations that need further investigation. For example, while we applied our method in Shenzhen City, we were unsure if it would work in other cities or regions. We focused on heavy metals; it remains to be seen if this method is effective for other soil properties. Additionally, using other multivariate analysis methods, such as deep learning, may result in different performance for nearby samples compared to PLSR. These limitations present opportunities for further study and analysis.

## 5. Conclusions

This study explored using spatially nearby samples to estimate soil Cu in urban areas with vis-NIR spectroscopy. Our results lead to the following conclusions: (i) Using spatially nearby samples significantly improves the Cu estimation model compared to traditional methods. (ii) As the number of nearby samples increases, the performance of the Cu estimation model follows an inverted U-shaped curve, initially increasing and then declining. (iii) The optimal number of nearby samples should strike a balance, covering a moderate area without there being too few or too many. We found that using 125 nearby samples, or 62.5% of the total calibration samples, is optimal.

We successfully used spatially nearby samples to estimate soil Cu with high accuracy. However, more research is still needed on estimating soil Cu, such as using machine learning methods. While our study focuses on soil heavy metals in urban soils, we should also explore how this approach works in agricultural land and other soil properties under different environmental conditions.

## Figures and Tables

**Table 1:** The descriptive statistics of 250 soil samples for the calibration and validation sets.

Sample	Number	Cu (mg·kg^−1^)									
		Min	Max	Median	Mean	Std ^1^	CV ^2^	Skewness	Kurtosis	Background	Pollution Level
**Total**	250	20.45	103.24	59.44	58.29	15.57	0.27	0.13	0.12	17.00	36.00
**Calibration**	200	20.45	103.24	59.44	58.29	15.60	0.27	0.13	0.15		
**Validation**	50	25.21	97.06	59.18	58.30	15.63	0.27	0.13	0.12		

^1^ Std denotes standard deviation. ^2^ CV denotes coefficient of variation.

**Table 2:** Summary statistics for soil Cu estimation models using spatially nearby samples.

Number of Nearby Samples	Calibration		Validation		
	$R_{cv}^{2}$	${RMSE}_{cv}$	$R_{p}^{2}$	RMSEP	RPD
None	0.64	9.72	0.75	8.56	1.83
20	-	-	0.75	7.75	2.01
50	-	-	0.90	4.90	3.14
100	-	-	0.92	4.56	3.43
150	-	-	0.93	4.00	3.90
200	-	-	0.92	4.34	3.60

Note: $R_{cv}^{2}$ denotes coefficient of determination in cross-validation. ${RMSE}_{cv}$ denotes root mean square error in cross-validation. $R_{p}^{2}$ denotes coefficient of determination in prediction. RMSEP denotes root mean square error of prediction. RPD denotes the residual predictive deviation. LV denotes latent variable.

## Data Availability

The original contributions presented in the study are included in the article; further inquiries can be directed to the corresponding author.
